# Safety and Efficacy of Trastuzumab Emtansine in Advanced Human Epidermal Growth Factor Receptor 2–Positive Breast Cancer: a Meta-analysis

**DOI:** 10.1038/srep23262

**Published:** 2016-03-16

**Authors:** Kai Shen, Xuelei Ma, Chenjing Zhu, Xin Wu, Hongyuan Jia

**Affiliations:** 1 State Key Laboratory of Biotherapy and Cancer Center, West China Hospital, Sichuan University, and Collaborative Innovation Center for Biotherapy, Chengdu 610041, PR China

## Abstract

Advanced or metastatic breast cancer is an incurable disease with high mortality rate worldwide and about 20% of breast cancers overexpress and amplify the human epidermal growth factor receptor 2 (HER2). Achievements in targeted therapy have benefited people during the past decades. Trastuzumab emtansine (T-DM1), a novel antibody-drug conjugate playing a powerful role in anti-tumor activity, not only blocks the HER2 signaling pathways, but also disturbs the microtubule dynamics. To access the efficacy and safety of T-DM1, we analyzed 9 clinical trials on T-DM1. Results showed that fatigue (0.604, 95% CI 0.551, 0.654), nausea (0.450, 95% CI 0.365, 0.537), increased transaminases (0.425, 95% CI 0.353, 0.500) and thrombocytopenia (0.383, 95% CI 0.322, 0.448) occurred more frequently in participants with single T-DM1. In controlled trials, increased transaminases (OR = 4.040, 95% CI 1.429, 11.427), thrombocytopenia (OR = 8.500, 95% CI 3.964, 18.226) and fatigue (OR = 1.288, 95% CI 1.041, 1.593) were statistically significant. Only thrombocytopenia appeared as severe adverse event (grade ≥ 3) in single-arm and control-arm studies. Meanwhile, T-DM1 stabilized cancer and prolonged life with notable improved progression-free survival (PFS) and overall survival (OS). In conclusion, it is a safe and effective agent in advanced or metastatic breast cancer, but should be carefully applied on patients with severe hepatic and neurological disease.

Breast cancer is one of the most common cancers among women worldwide^1^. It is the second leading cause of cancer death among women in the U.S, exceeded only by lung cancer^2^. With the advancements of chemoradiotherapy over the past two decades, the prognosis of breast cancer has improved and the 5-year overall survival rate is almost up to 90%^3^. However, metastatic breast cancer (MBC) remains a challenge with only about 22 month’s overall survival (OS)^1^. According to the expressions of estrogen, progesterone and human epidermal growth factor receptor 2 (HER2), breast cancers were traditionally divided into four types^4^. HER2 is overexpressed and amplified in about 20% of all breast cancers^3^,^4^ and functions as a poor prognostic factor^5^. Blockage of HER2 receptors has attracted much attention and in 1998 the US Food and Drug Administration (FDA) approved trastuzumab as an agent in HER2-positive breast cancer therapy^6^. However, some patients who were treated with trastuzumab still appeared disease progression and trastuzumab resistance limited the clinical application of trastuzumab. In 2013, the FDA approved the application of trastuzumab emtansine (T-DM1) on patients with trastuzumab resistance^7^,^8^.

T-DM1 is a novel antibody-drug conjugate composed of trastuzumab, derivative of maytansine 1 (DM1) and a non-reducible thioether linker^9^,^10^. Trastuzumab itself is a humanized monoclonal antibody targeting on HER2 receptors, thus stimulating antibody-dependent cell-mediated cytotoxicity (ADCC), inhibiting the PTEN-PI3K/AKT pathway and inducing apotosis^3^,^11^,^12^,^13^. Moreover, after T-DM1 binds to HER2, the HER2-T-DM1 complex enters into target cells through receptor-mediated endocytosis and releases DM1, causing cell cycle arrest and apoptosis via the inhibition of microtubule assembly^11^,^12^. To access the efficacy and safety of T-DM1, clinical trials were launched in different countries among patients with advanced or metastatic HER2-positive breast cancer. Studies have demonstrated that T-DM1 functioned well in patients. In a phase 1 trial, researchers^14^ have detected the maximum-tolerated dose and the optimal outcome was 3.6 mg/kg every three weeks. A large-scale trial^15^ comparing T-DM1 with lapatinib plus capecitabine among HER2-positive metastatic breast cancer patients indicated that T-DM1 significantly improved the OS and median progression-free survival (PFS). T-DM1 brought clinical benefits to patients, at the same time, the adverse events (AEs) were inevitable. The most common AEs caused by T-DM1 were fatigue, nausea, increased aminotransferases, thrombocytopenia, arthralgia, headache, anemia and pneumonia. Thus, we analyzed published clinical trials to evaluate the odd ratios (ORs) of adverse events and hazard ratios (HRs) for PFS and OS.

## Results

### Eligible articles

**Figure 1:**
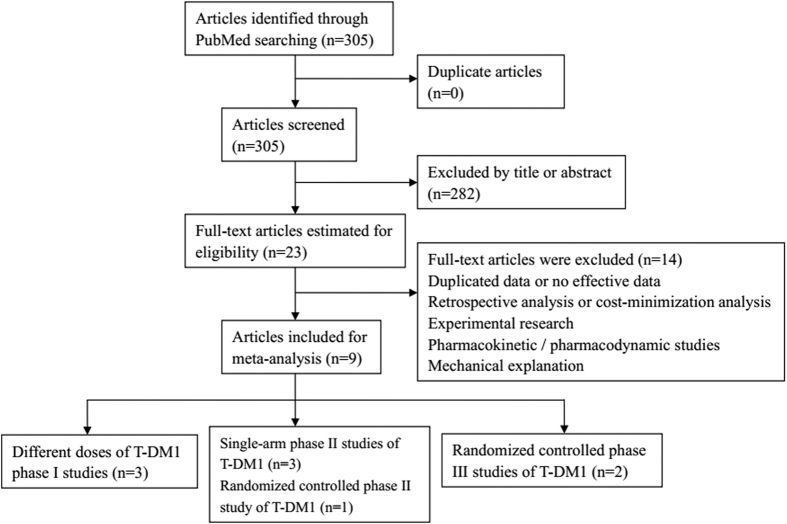
Flow diagram of the literature search and trial selection process.

A total of 305 potentially relevant articles were searched in PubMed in June 2015. After reviewing the titles or abstracts, 282 unrelated articles were excluded. After reading the full texts of the remaining articles, 9 articles were finally included for meta-analysis. The basic process was showed in Fig. 1. Basic information of the 9 included articles was presented in Table 1. Eligible articles included 3 phase I trials and 3 phase II single-arm studies, and 1 phase II and 2 phase III studies were randomized controlled trials. For single-arm trials, we calculated the incidence of adverse events and analyzed 3 articles for PFS. In terms of controlled studies, Hurvitz *et al.* set two groups (T-DM1; trastuzumab plus docetaxel) to determine the contribution of T-DM1 in adverse events, Verma *et al.* made comparison between Lapatinib plus Capecitabine and T-DM1, Krop *et al.* conducted a multicenter trials comparing T-DM1 and physician’s choice of treatment, both adverse events and efficacy were analyzed.

### Patients

A number of 2050 patients (T-DM1: 1308; control: 742) in 9 articles were included for analysis. For safety analysis, Hurvitz *et al.* excluded two patients from their study and included another two patients taking T-DM1 in control arm (T-DM1: 1310; Control: 738). Three trials (T-DM1: 962; Control: 738) compared T-DM1 with other agents. Miller *et al.* used T-DM1 and pertuzumab for single-arm study (n = 64). The others (n = 348) took single T-DM1 for therapy.

### Safety analysis

All the included articles reported adverse events. After evaluating the all grade and grade ≥3 adverse events, we found that the most common events contained anemia, fatigue, increased transaminases, nausea, thrombocytopenia, arthralgia and headache. Single-arm studies were analyzed to calculate the adverse event rates and control trials were to determine the contribution of T-DM1 in adverse events. The results were presented in Table 2.

**Figure 2:**
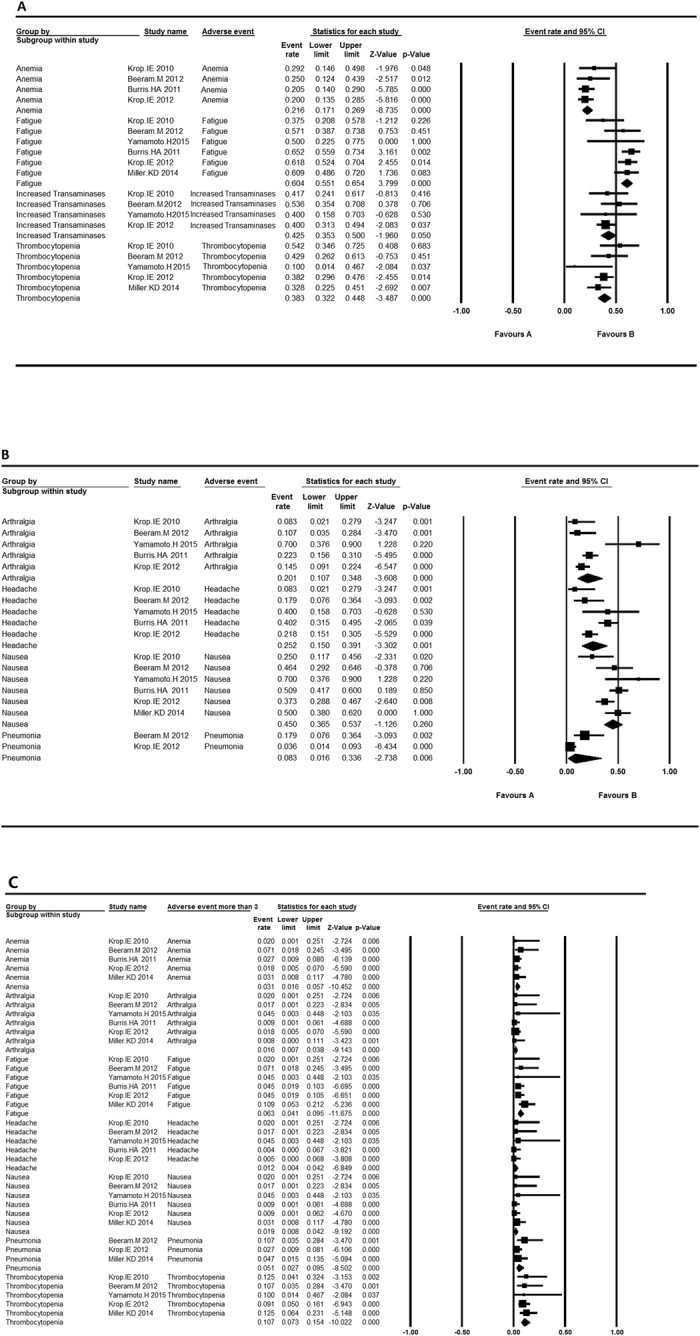
The adverse event rates and 95% CI in single-arm trials. (**A**) The adverse event rates and 95% CI of fixed model in single-arm trials; (**B**) The adverse event rates and 95% CI of random model in single-arm trials; (**C**) The adverse event (grade more than 3) rates and 95% CI of fixed model in single-arm trials.

With regard to single-arm trials (Fig. 2), the incidence of anemia ranged from 0.200 to 0.292 and the overall event rate accounted for 0.216 (95% CI 0.171, 0.269). Increased transaminases occurred more frequently in participants with the overall event rate being 0.425 (95% CI 0.353, 0.500). Thrombocytopenia showed a similar event rate of 0.383 (95% CI 0.322, 0.448). Nausea appeared differently in each trial, varying from 0.250 to 0.700 and the overall event rate was 0.450 (95% CI 0.365, 0.537). Fatigue was the most common adverse event with the highest rate of 0.604 (95% CI 0.551, 0.654). Arthralgia and headache occurred less frequently. Despite the high rates of adverse events, severe events (grade ≥ 3) were relatively rare. Only severe thrombocytopenia occurred in 10.7% (95% CI 0.073, 0.154) of participants, others seldom happened.

**Figure 3:**
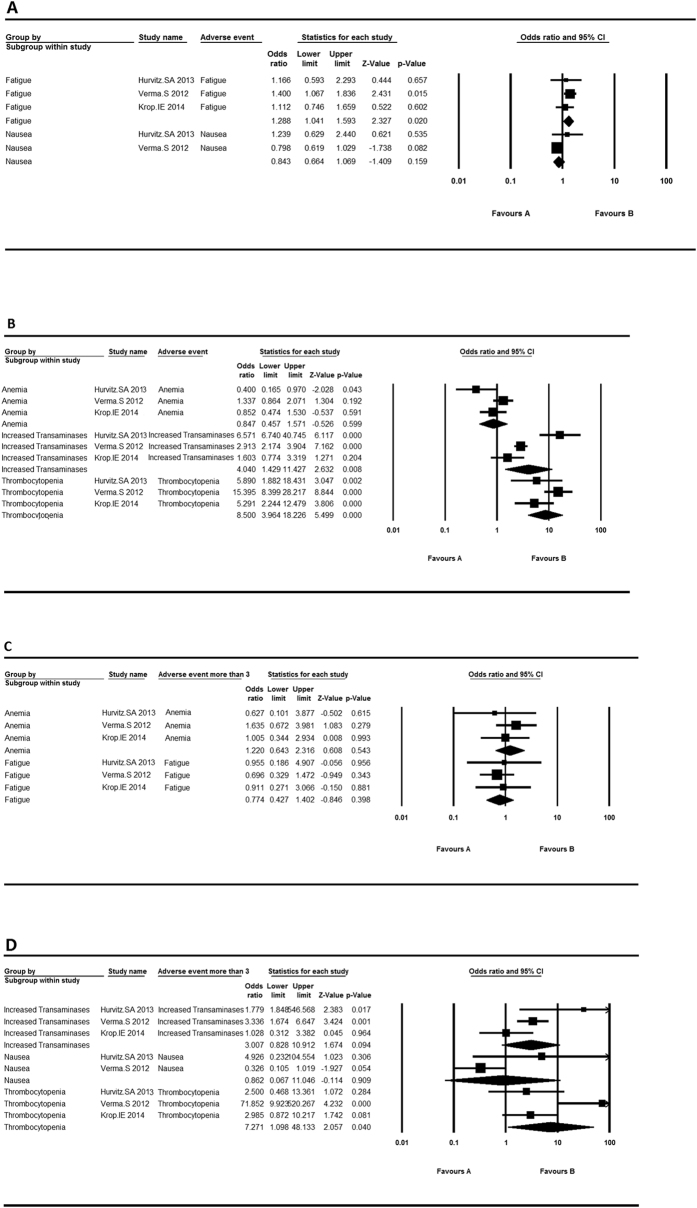
The adverse event rates and 95% CI in control-arm trials. (**A**) The adverse event rates and 95% CI of fixed model in control-arm trials; (**B**) The adverse event rates and 95% CI of random model in control-arm trials; (**C**) The adverse event (grade more than 3) rates and 95% CI of fixed model in control-arm trials; (**D**) The adverse event (grade more than 3) rates and 95% CI of random model in control-arm trials.

For three control-arm studies, we analyzed anemia, fatigue, increased transaminases, nausea and thrombocytopenia with OR values (Fig. 3). According to I^2^, fixed model was used in all grade fatigue and nausea, and grade ≥3 anemia and fatigue. Among all the adverse events, increased transaminases (OR = 4.040, 95% CI 1.429, 11.427), thrombocytopenia (OR = 8.500, 95% CI 3.964, 18.226) and fatigue (OR = 1.288, 95% CI 1.041, 1.593) were statistically significant. T-DM1 may play a dominant role in thrombocytopenia with the highest OR value. Similarly, only grade ≥3 thrombocytopenia (OR = 7.271, 95% CI 1.098, 48.133) appeared statistically significant.

### Efficacy analysis

**Figure 4:**
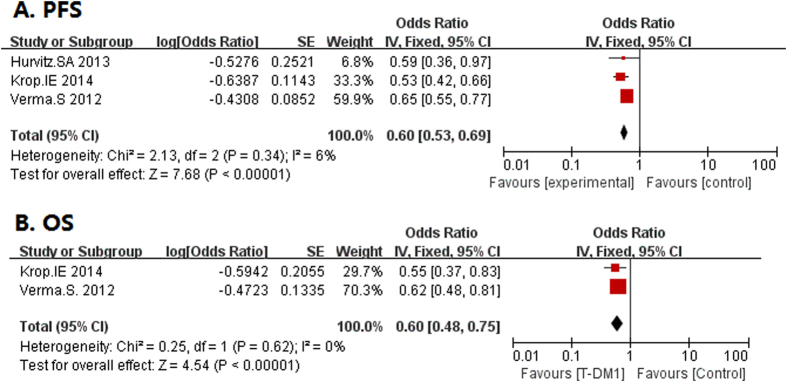
The HRs and 95% CI for PFS and OS in control-arm trials. (**A**) PFS; (**B**) OS.

PFS data was presented in 3 single-arm studies and 3 controlled trials (Table 3). The median PFS varied from 4.6 to 6.9 months for single-arm studies. In controlled trials, the HRs for progression or death in three control trials ranged from 0.528 to 0.65 with a total OR of 0.64 (95% CI 0.55, 0.75) (Fig. 4), indicating a longer PFS in T-DM1 group. Only two controlled studies provided the OS data, with a total OR of 0.64 (95% CI 0.55, 0.75) (Fig. 4). Verma *et al.* showed both first and second interim analysis and Krop *et al.* only had second interim analysis, both indicating an improved survival than other groups.

## Discussion

Although the advanced or metastatic breast cancer remains incurable, the application of T-DM1 does benefit patients. According to our analysis, major common adverse events involved fatigue, nausea, increased transaminases and thrombocytopenia. The total event of fatigue reached more than 50% and nausea happened in nearly half of participants. The OR value of increased transaminases was more than 3, indicating the firm correlation with the toxicity of T-DM1. Compared to this, the event rate of anemia and thrombocytopenia seemed lower, but severe thrombocytopenia (grade ≥ 3) approached 10% and the OR valued more than 5, suggesting a prominent role of T-DM1 in hematologic toxicity. Other adverse events did not show such close correlations with T-DM1. Meanwhile, we summarized the PFS and OS in patients. As was shown in single-arm trials, T-DM1 stabilized the disease approximately for half a year. In controlled trials, T-DM1 was more effective than other therapies, even compared with the combination therapy with trastuzumab^16^. T-DM1 indeed brings hope and benefits to patients.

As described above, the components trastuzumab and DM1 both play a role in anti-tumor activity. Trastuzumab (Herceptin) is a humanized IgG antibody specific to HER2, which was approved for HER2-positive breast cancer therapy. It is reported to activate the tumor suppressor PTEN, down-regulate the ErbB2 and subsequently inhibit PTEN-PI3K/AKT signaling pathway, which is vital for diverse cell functions including cell growth, survival, proliferation and metabolism^17^,^18^,^19^,^20^. In T-DM1, trastuzumab not only functions as an antibody binding to the HER2-positive cells, inhibiting the HER2 signaling pathway and inducing antibody-mediated cellular cytotoxicity (ADCC), but also specifically conveys DM1 to target cells which disturbs the original cell function^9^,^21^. An experiment^22^ has reported that the major toxicities of T-DM1 were associated with DM1 rather than trastuzumab or thioether linker. DM1 is a tubulin-binding agent. Once separated from ADCs, free DM1 has a high affinity to the microtubules, thus suppressing microtubule dynamics and inhibiting mitosis at metaphase^23^,^24^. Combination of the two powerful anti-tumor agents with a thioether linker makes it possible to function effectively in advanced or metastatic breast cancer and minimize the exposure of DM1 to normal tissue owing to the stable linker^25^.

The DM1 part in T-DM1, a microtubule-inhibiting agent, plays a major role not only in anti-tumor activity, but also in adverse events. Fatigue is the most common adverse event mainly attributed to DM1. Fortunately, few patients experienced severe fatigue. Previous studies have demonstrated that microtubule-inhibiting chemotherapy agents are always accompanied by neurotoxicity and DM1 is no exception^26^, DM1 or T-DM1 shares the same mechanism that causes notable degeneration of axon in animal experiments and may be less reversible^22^,^26^. Patients with nerve neurological problems should be cautious when taking T-DM1.

T-DM1 was given intravenously at 3.6 mg/kg every three weeks. Krop *et al.*^27^ confirmed the maximum tolerated dose of T-DM1 mainly according to severe thrombocytopenia (grade ≥ 3). Thrombocytopenia might result from decreased production or accelerated destruction of platelet. Among T-DM1 treated patients, researchers found that T-DM1 inhibited the differentiation of megakaryocytes and the production of platelets was consequently reduced^22^,^26^. Moreover, Uppal *et al.*^28^ reported that T-DM1 entered the megakaryocytes (MKs) by binding to FcgRIIα independent of HER2, and affected the cytoskeleton of differentiating MKs without trastuzumab. Another trial found a significantly positive relationship between HER2 expression and increased platelets through vascular endothelial growth factor (VEGF)^29^, but whether thrombocytopenia was caused by decreased HER2-positive breast cancer cells still remained unknown. More subsequent researches are needed to explore the exhaustive mechanism.

A previous study^26^ has confirmed that increased transaminases was caused by maytansine, and FDA has taken hepatotoxicity into account which predicts liver damage in people based on monkey experiments. In addition, other researches have found that the clearance of T-DM1 mainly depend on hepatic-biliary and gastrointestinal route^30^,^31^,^32^, hence patients with hepatic diseases should be kept under surveillance.

The article analyzed the safety and efficacy of T-DM1 in available clinical trials. All eligible articles chose patients with advanced or metastatic breast cancer. Possible mechanisms of major adverse events were explained. The heterogeneity of included articles was analyzed based on different regions, different races, different therapies and previous treatments, even the Eastern Cooperative Oncology Group performance status (ECOG PS) was analyzed.

There are also some shortages in our analysis. Firstly, Brain-metastatic breast cancer markedly influences PFS and OS, but in all articles, analysis of subtypes and stages of breast cancer were ignored, so we did not consider these aspects. Secondly, some articles mentioned that T-DM1 had two black boxes, one for pregnant women, the other for cardiac toxicity^32^. For trastuzumab part, it has been reported to cause cardiac dysfunction^33^, but cardiac AEs did not appear to be such frequent or serious in T-DM1, thus we did not take it into account, and further trials are needed. Trastuzumab has been reported to cause severe AEs in both pregnant women and fetuses^32^, but we found no descriptions of such case in T-DM1. Lastly, Miller *et al.*^34^ prescribed T-DM1 and pertuzumab in patients, but there was no consideration of the AEs of pertuzumab and the interactions between the two drugs in the analysis. All of these might cause bias in our analysis.

To sum up, T-DM1 is a relatively safe and effective agent in the treatment of advanced or metastatic HER2-positive breast cancer, even among patients with asymptomatic or treated brain metastases and trastuzumab resistance^14^,^15^,^16^,^25^,^27^,^34^,^35^,^36^,^37^. Previous articles^32^,^33^,^38^,^39^ found the same common AEs in trials including increased transaminases, thrombocytopenia and fatigue, but they had no explanations about them and further researches are needed. Given the notable adverse events in platelet production and drug excretion pathway, patients should take regular laboratory examination and should be followed up. For patients with severe hepatic or neurological diseases, drugs should be taken under close surveillance or should not be prescribed.

## Method

### Article searching

Relevant articles were selected by searching databases through PubMed (until June 2015) without language or data limitations. Retrieval keywords included “T-DM1”, “TDM-1”, “trastuzumab emtansine/trastuzumab-emtansine”, “kadcyla”, “ado-trastuzumab emtansine” and “trastuzumab-DM1”. The search was focused on articles conducting clinical trials.

### Inclusion and Exclusion criteria

The eligible criteria included: 1) any phase clinical trials evaluating the efficacy and safety of T-DM1 whether they had control groups or not; 2) patients in clinical trials were confirmed by pathology to have breast cancer, clinical evidence supported advanced or metastatic breast cancer; 3) efficacy and adverse events were available in the results; 4) full text could be downloaded. Articles were excluded if they were duplicate publications or without raw data.

### Data extraction

Data extracted from all eligible articles included: 1) the basic information of studies: the first author name, year of publication, study design, number of participants, treatment and study phase. 2) the characteristics of major AEs (mentioned in at least 2 articles): T-DM1 groups or control groups, types of AEs and numbers of all grade and grade ≥3 AEs. 3) HRs for PFS or OS.

### Statistical analysis

Data analysis was performed on Comprehensive Meta-Analysis (CMA) program 2 (Biostat, Englewood, NJ) and Review manager 5.2 (Copenhagen, Sweden). For single-arm studies, we calculated the proportion and derived 95% confidence interval (CI) of major AEs (both all grade and grade ≥3). For controlled trials, the OR was calculated to determine the role of T-DM1 in adverse events. Two-sided P values were considered significant when less than 0.10 and I^2^ ≥ 50% was used to decide on fixed-effects model or random-effects model in the analysis.

## Additional Information

**How to cite this article**: Shen, K. *et al.* Safety and Efficacy of Trastuzumab Emtansine in Advanced Human Epidermal Growth Factor Receptor 2-Positive Breast Cancer: a Meta-analysis. *Sci. Rep.* **6**, 23262; doi: 10.1038/srep23262 (2016).

## Figures and Tables

**Table 1:** Basic information of eligible articles.

First author	Year of publication	phase	treatment	Number of patients
Krop.IE	2010	I	T-DM1	24
Beeram.M	2012	I	T-DM1	28
Yamamoto.H	2015	I	T-DM1	10
Burris.HA 3rd	2011	II	T-DM1	112
Krop.IE	2012	II	T-DM1	110
Hurvitz.SA	2013	II	T-DM1	137(67,70)
Trastuzumab plus docetaxel				
Miller.KD	2014	Ib/IIa	T-DM1 and pertuzumab	64
Verma.S	2012	III	T-DM1	991(495; 496)
Lapatinib plus capecitabine				
Krop.IE	2014	III	T-DM1	602(404; 198)
Physician’s choice				

**Table 2:** The OR values and models of control-arm and the event rates of single-arm trials.

Control-arm trials:						
Adverse events	All grade			Grade ≥3		
	Odds Ratio with 95% CI	Model	I^2^	Odds Ratio with 95% CI	Model	I^2^
Anemia	0.847(0.457,1.571)	Random Model	67.184	1.220(0.643,2.316)	Fixed Model	0.000
Fatigue	1.288(1.041,1.593)	Fixed Model	0.000	0.774(0.427,1.402)	Fixed Model	0.000
Increased Transaminases	4.040(1.429,11.427)	Random Model	87.995	3.2007(0.828,10.912)	Random Model	65.316
Nausea	0.843(0.664,1.069)	Fixed Model	29.670	0.862(0.067,11.046)	Random Model	62.427
Thrombocytopenia	8.500(3.964,18.226)	Random Model	59.033	7.271(1.098,48.133)	Random Model	75.811
Single-arm trials:						
Adverse events	Event rate with 95% CI					
Fixed model						
Anemia	0.216(0.171,0.269)					
Fatigue	0.604(0.551,0.654)					
Increased Transaminases	0.425(0.353,0.500)					
Thrombocytopenia	0.383(0.322,0.448)					
Random model						
Arthralgia	0.201(0.107,0.348)					
Headache	0.252(0.150,0.391)					
Nausea	0.450(0.365,0.537)					
Pneumonia	0.083(0.016,0.336)					

**Table 3:** The PFS of control-arm trials and single-arm trials.

Control-arm trials:				
study	PFS(median month)		HR(95% CI)	P value
	T-DM1	Control		
Hurvitz.SA 2013	14.2	9.2	0.59(0.36,0.97)	0.035
Verma.S 2012	9.6	6.4	0.65(0.55,0.77)	<0.001
Krop.IE 2014	6.2	3.3	0.528(0.422,0.661)	<0.0001
Single-arm trials				
Study	Median PFS(95% CI)(month)			
Burris.HA 3rd 2011	4.6(3.9,8.6)			
Krop.IE 2012	6.9(4.2,8.4)			
Miller.KD 2014	6.6(4.21,9.46)			
